# X-ray nanoprobes and diffraction-limited storage rings: opportunities and challenges of fluorescence tomography of biological specimens

**DOI:** 10.1107/S160057751401621X

**Published:** 2014-08-27

**Authors:** Martin D. de Jonge, Christopher G. Ryan, Chris J. Jacobsen

**Affiliations:** a Australian Synchrotron, 800 Blackburn Road, Clayton, Victoria 3168, Australia; b CSIRO Earth Science and Research Engineering, Clayton, Victoria 3168, Australia; c Advanced Photon Source, Argonne National Laboratory, 9700 South Cass Avenue, Argonne, IL 60439, USA; d Department of Physics, Chemistry of Life Processes Institute, Northwestern University, 2170 Campus Drive, Evanston, IL 60208, USA; e Chemistry of Life Processes Institute, Northwestern University, 2170 Campus Drive, Evanston, IL 60208, USA

**Keywords:** X-ray fluorescence microscopy, fluorescence tomography, nanotomography, scanning X-ray microscopy, diffraction-limited storage rings

## Abstract

X-ray nanoprobes require coherent illumination to achieve optic-limited resolution, and so will benefit directly from diffraction-limited storage rings. Here, the example of high-resolution X-ray fluorescence tomography is focused on as one of the most voracious demanders of coherent photons, since the detected signal is only a small fraction of the incident flux. Alternative schemes are considered for beam delivery, sample scanning and detectors. One must consider as well the steps before and after the X-ray experiment: sample preparation and examination conditions, and analysis complexity due to minimum dose requirements and self-absorption. By understanding the requirements and opportunities for nanoscale fluorescence tomography, one gains insight into the R&D challenges in optics and instrumentation needed to fully exploit the source advances that diffraction-limited storage rings offer.

## Abstract 2

Nanoscale X-ray scanning microscopes, or X-ray nanoprobes, will benefit greatly from diffraction-limited storage rings. Here the requirements for nanoscale fluorescence tomography are explored to gain insight into the scientific opportunities and technical challenges that such sources offer.

## 1. Introduction   

Diffraction-limited storage rings offer a dramatic increase in beam brightness from a high-repetition-rate source. While the energy per pulse in X-ray free-electron lasers often causes immediate thermal and ionizing damage when the beam is tightly focused, storage rings provide a higher frequency of pulses with individually lower energy, so that a single unique sample can be explored from many directions and/or with many photon energies. As a result, these two source types serve complementary roles.

The dramatic increase in source brightness offered by diffraction-limited storage rings translates directly into coherent flux. It is therefore natural to think of coherent imaging and scattering experiments such as photon correlation spectroscopy or coherent diffraction imaging as the most logical beneficiaries of this advance in storage ring light sources. While X-ray fluorescence is an incoherent process (meaning that there is no wave correlation between a photon absorbed by an atom and a photon emitted later *via* fluorescence), most X-ray fluorescence microscopes are scanning microscopes, and scanning microscopes can employ only the coherent fraction of the beam if one wishes to focus the beam to the diffraction limit of an aberration-free optic. As a result, any nanoprobe experiment that involves a lower intensity indirect signal such as fluorescence (or inelastic scattering, which is not discussed here) will directly benefit from the increases in source brightness that diffraction-limited storage rings can offer. This is important for scanning X-ray fluorescence microscopy (XFM), and it is especially important for its three-dimensional variant of X-ray fluorescence tomography.

We discuss here the scientific importance of X-ray fluorescence tomography with an emphasis on biological applications, some aspects of beamline and experimental design, and potential capabilities that a diffraction-limited storage ring can enable.

## 2. X-ray fluorescence tomography of biological specimens   

Advances in synchrotron X-ray sources and in microscope optics and instrumentation have led to an ever-increasing impact of XFM in biomedical research. At a recent FASEB summer research conference on Trace Element Metabolism in Biology and Medicine (Steamboat Springs, June 2012 ▶), roughly a quarter of the talks included data from X-ray microprobes; an August 2013 workshop on XFM at Northwestern’s Feinberg School of Medicine drew 80 international participants. A growing number of reviews and opinion articles echo this trend (Paunesku *et al.*, 2006 ▶; Fahrni, 2007 ▶; Szczerbowska-Boruchowska, 2008 ▶; de Jonge & Vogt, 2010 ▶; Fittschen & Falkenberg, 2011 ▶; Majumdar *et al.*, 2012 ▶). Recent example studies include the imaging of TiO_2_-DNA nanocomposites that can be used for intracellular gene targeting in possible cancer therapy approaches (Paunesku *et al.*, 2007 ▶). Follow-on studies have added fluorescence imaging of gadolinium magnetic resonance imaging (MRI) labels which can be conjugated onto the same composites (Paunesku *et al.*, 2008 ▶); this allows one to understand the subcellular sequestration of the very same contrast agents that are used in MRI to study organs and tumors. In a different study, Malinouski *et al.* used XFM to visualize trace element selenium in mammalian tissues; they found a highly localized pool of this trace element at the basement membrane of kidneys, and were able to demonstrate its association with the protein glutathione peroxidase 3 (Malinouski *et al.*, 2012 ▶), while Weekly *et al.* used XFM to study the metabolism of selenite in human lung cancer cells (Weekley *et al.*, 2011 ▶).

X-ray fluorescence microscopy also plays an important role in plant research. Metals can play important roles in natural plant processes such as that of Mn in photosynthesis, or disruptive roles such as that of Cr in plant growth. Example studies using fluorescence tomography include understanding the localization of Ni and Mn in the nickel hyperaccumulating plant *Alyssum murale*, showing that Ni is removed from the soil by fine roots and distributed through the plant leaf into dermal tissues and trichome bases (McNear *et al.*, 2005 ▶). Several recent reviews highlight how X-ray fluorescence nanotomography provides important information when coupled with plant molecular biology (Donner *et al.*, 2012 ▶) or X-ray absorption spectroscopy (Gräfe *et al.*, 2014 ▶), thus highlighting the importance of three-dimensional measurements in the broader context of the use of synchrotron radition for studies of the role metals play in plant metabolism (Sarret *et al.*, 2013 ▶).

**Figure 1:**
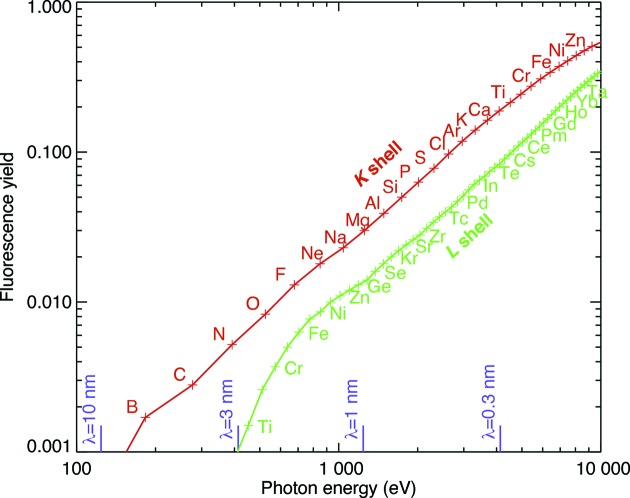
X-ray fluorescence yield of the naturally occurring elements (Krause, 1979 ▶) as a function of the energy of the emitted photon. To detect fluorescence from a given element, one must excite the atom through absorption of a photon with an energy sufficient to eject an electron from a given X-ray ‘shell’ or atomic orbital. The process that competes with fluorescence emission, Auger electron emission, is not well suited to elemental detection in thick biological specimens because Auger electrons tend not to escape at their original element-specific energy except from regions within about 100 nm of the sample’s surface. While notable success has been achieved in X-ray fluorescence microscopy of light elements (Kaulich *et al.*, 2009 ▶), their low fluorescence yield and strong self-absorption make detection difficult with consequently higher radiation dose imparted.

The penetrating power of X rays allows one to image many-micrometer-thick specimens in a way that electron microscopes cannot. While soft X-ray nanotomography systems allow visualization of major subcellular structures in few-micrometer-thick specimens (Schneider *et al.*, 2002 ▶, 2010 ▶; Larabell & Le Gros, 2004 ▶), hard or ∼5–20 keV X-ray microscopes are able to excite X-ray fluorescence from most biologically significant metals (Fig. 1 ▶) even at trace concentrations, as well as offering improved depth of field for a given value of the transverse resolution 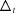 (see §4.1). In electron microprobes, fluorescent X-rays sit atop a large continuum X-ray background (LeFurgey & Ingram, 1990 ▶), while in XFM this background is largely absent (the relevant scattering background is many orders of magnitude lower than the bremsstrahlung background present with electron excitation). The result is that XFM offers higher sensitivity with many orders of magnitude less radiation damage compared with electron or proton microprobes (Kirz, 1980*b* ▶; Sparks, 1980 ▶). By using a single beam energy above all absorption edges of interest, and using an energy-resolving detector, one can detect many elements simultaneously in one measurement. These capabilities nicely complement the high-spatia-resolution capabilities of electron microprobes for studies of thin sections. XFM also complements the live cell imaging abilities of fluorescence light microscopy, where absolute quantitation of metal content requires exact knowledge of the different binding affinities of fluorophores in all of the cell’s biochemical compartments; fluorophores are available for only a subset of interesting trace metals, and may reveal only certain ionic and chemical forms of trace metals.

The simplest approach for nanoscale X-ray fluorescence microscopy would be to have an imaging optic collect the fluorescence from all elements over a large field of view and deliver the image onto an energy-sensitive pixelated area detector. However, while reflective optics are achromatic, aberrations and acceptance angles limit their use as field imaging optics so they tend to be used mainly for producing focused beam spots. Optics such as Fresnel zone plates and compound refractive lenses have been used with great success as field-imaging optics for monochromatic light, but they have dispersion that scales as λ and λ^2^, respectively, so there is not a straightforward way to use them for imaging multiple fluorescence lines simultaneously. In addition, available pixelated detectors with energy resolution involve either too few pixels for practical imaging (for example, the 384 pixels in the case of the Maia detector discussed in §4.5) or a requirement that there be no more than one photon per pixel per ∼1 ms readout time if one wants to measure the energy of individual photons. This latter case applies to energy-resolving CCD detectors such as the pnSensor CCD camera which shows great success for ∼20 µm fluorescence imaging using X-ray tubes and collimating optics (Ordavo *et al.*, 2011 ▶). However, we do not see a path to full-field fluorescence imaging of multiple elemental signals at the nanoscale.

**Figure 2:**
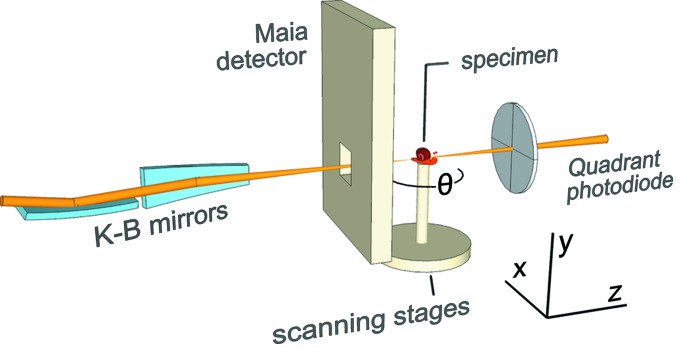
Schematic of the X-ray fluorescence microprobe instrument at the Australian Synchrotron XFM beamline (Paterson *et al.*, 2011 ▶). A monochromated X-ray beam is focused to a small spot using a Kirkpatrick–Baez (K–B) mirror pair. The sample is scanned and rotated through this focus. A segmented transmission detector records the transmitted intensity and position to obtain differential phase contrast images (de Jonge *et al.*, 2008 ▶), and the energy-resolving Maia detector system (Kirkham *et al.*, 2010 ▶) records an event stream including X-ray energy and specimen position information.

Because of these considerations, X-ray fluorescence microscopy tends to be done using scanning microscopy. The probe defines the spatial resolution, and an energy-dispersive or wavelength-dispersive detector measures the energy of photons emitted from each illuminated spot (Fig. 2 ▶). The overall sensitivity of elemental mapping is determined in part by the detector and the experimental geometry. The Maia detector depicted in this figure covers an extremely large solid angle (∼1.2 sr), and is used in the backscatter geometry to optimize experimental efficiency for large specimens such as paintings (Howard *et al.*, 2012 ▶), polished mineral sections (Ryan *et al.*, 2010*b* ▶) and large areas of cells (James *et al.*, 2013*b* ▶) (this and other detector geometries are discussed in §4.5). Scanning allows imaging of arbitrary field sizes limited only by the nanopositioning system, and simultaneous acquisition of multiple signal modes as will be described in §4.6. Unlike the case in scanning light or electron microscopy where it is easier to scan the probe beam, in X-ray microscopes the sample is usually scanned (though a speculative X-ray beam scanning approach will be shown in Fig. 5).

**Figure 3:**
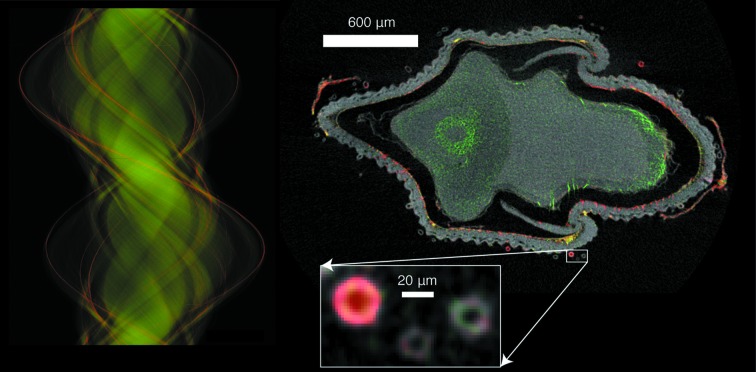
High-definition X-ray fluorescence tomography enables virtual sectioning to expose the internal distribution of particular elements. Shown here is a single-slice X-ray tomogram of an immature rice grain that has been pulsed with germanic acid to mimic the uptake of arsenite by rice. It is generally known that arsenite enters through the silicic acid pathway; by using Ge as a proxy for Si, (inferred) silicic and arsenite distributions can be mapped nondestructively and at high resolution to further understand transport mechanisms (Carey *et al.*, 2011 ▶). At left is the sinogram, a projection line from a single height along the rotation axis, shown as a function of rotation angle (the name comes from the fact that off-axis features trace a sine wave as the sample is rotated). Standard tomographic reconstruction yields the two-dimensional image shown at right, which is a tomographic ‘slice’ of the object at that height along the rotation axis with Ge concentration shown in red, Zn concentration in green, and overall mass as estimated from Compton scattering shown in shades of gray. These data were acquired using the X-ray fluorescence microprobe beamline at the Australian Synchrotron (Fig. 2 ▶); the measurement covered a 4.6 mm scan width at a resolution of 2 µm and a per-pixel transit time of ∼1.9 ms, so that acquisition of projections over 2000 angular steps over a 360° range (4.6 Mpixel) took less than 3 h. By collecting data over a 360° angular range rather than 180°, self-absorption effects could be gauged; these were found to be minimal for Ge and moderate for Zn, while lighter elements were strongly affected (not shown here). The inset shows a detail of the small hairs that grow on the outside of the husk.

Two-dimensional elemental imaging by X-ray fluorescence microscopy is extremely powerful, as described in the many reviews cited above. It allows one to study thin sections, or adherent cells on flat supports, and obtain reliable information on areal content of different elements. Correlative scanned-beam imaging techniques (§4.6) such as X-ray phase contrast (Hornberger *et al.*, 2007 ▶; de Jonge *et al.*, 2008 ▶; Holzner *et al.*, 2010 ▶; Kosior *et al.*, 2012 ▶) or ptychography (Vine *et al.*, 2012 ▶) can be used to determine projected mass, which (when coupled with assumptions of the density of the main consituents of the specimen, or the specimen ‘matrix’) allow one to go from areal content to projected volume concentration. Alternately, one can add measurements of the specimen’s thickness using a method such as atomic force microscopy (Lagomarsino *et al.*, 2011 ▶), and thus obtain projected elemental concentration directly. Elemental concentration is of great importance, because concentration gradients drive diffusion-based processes and thus play an important role in the biochemical function of cells. However, while projected concentrations provide additional insight, concentration gradients are three-dimensional. Further, cells are not two-dimensional objects, and if one wishes to determine whether nanoparticles used for drug delivery have arrived inside the nucleus (*versus* simply lying above or below the nucleus when viewed along a particular projection direction), three-dimensional imaging is necessary (Yuan *et al.*, 2013 ▶). Simply put, most real-life specimens are organized in three dimensions (see, for example, Fig. 3 ▶), so X-ray fluorescence tomography may be required for a complete picture of the role of various elements in the function of natural and manufactured materials.

## 3. Nanoprobes, beamlines and diffraction-limited storage rings   

As noted in the *Introduction* [Sec sec1], X-ray fluorescence is an inherently incoherent measurement process; why then might X-ray fluorescence tomography be dramatically improved through the use of a diffraction-limited storage ring? The answer lies in the formation of a nanoscale X-ray probe beam. Any lens used to form such a probe has a beam size limited by the lens’ aperture; this is usually expressed in terms of the Rayleigh resolution of 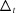 = 0.61λ/NA, where NA is the numerical aperture or sinus of the semi-opening angle of the optic when the medium between the optic and the focus has a refractive index of 1. The Rayleigh resolution can be exceeded by a modest amount, at a cost of loss of contrast for finer features (Jacobsen *et al.*, 1992 ▶); it measures the distance from the focus center to the first minimum of the Airy function of a circular aperture (Born & Wolf, 1999 ▶), so that the full width of the beam can be approximated by 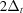. The product of the focal waist and the beam opening angle is equivalent to the phase space area occupied by a Hamiltonian system, and Liouville’s theorem states that this phase space cannot be decreased without performing work on the system, which is difficult in the case of photons! The phase space area of the diffraction-limited focus of a lens can be characterized by the full-width size of the focus times the accepted angle, leading to a full-width full-angle (FWFA) product 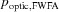 of 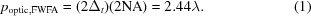
In fact, by simply convolving the geometrical image of a radiation source with the diffraction-limited focus of an optic, one arrives at the conclusion that the FWFA acceptance from the source should be kept to 0.5–1λ in each transverse direction, after which one can accept a larger phase space area from the beam with a gain in higher flux but a cost in reduced resolution (Winn *et al.*, 2000 ▶). This then leads to the central argument for the benefit of diffraction-limited sources for X-ray fluorescence tomography, or indeed any type of scanning microscopy: to achieve a focus limited by the optic rather than the source, one can only accept light from a phase space area of about λ in each transverse direction.

We note that advances in microscopy with visible-light fluorophores have gone far beyond the Rayleigh resolution limit (Hell, 2007 ▶). In stochastic imaging approaches, one measures the position of the centroid of a single fluorophore’s blurred image to high resolution (Betzig *et al.*, 2006 ▶; Rust *et al.*, 2006 ▶), while stimulated emission depletion methods can allow for sharpening of a fluorophore’s spatial response (Hell & Wichmann, 1994 ▶). However, the limitations of full-field fluorescence imaging complicate the former approach (§2), while radiation damage (§4.3) would seem to limit the latter approach to X-ray imaging. At present we do not see a practical way to go much beyond the Rayleigh resolution limit in X-ray fluorescence microscopy.

How then has research using nanofocused beams been able to take place prior to the development of diffraction-limited storage rings? Very simply, at the cost of flux. By placing an aperture at the position of an intermediate focus in a beamline (such as an exit slit in a focusing monochromator), or by placing a lens in a position where it accepts only a fraction of the light from the source, one can limit the phase space acceptance of the nanofocusing lens to ∼1λ. One way to think of this with a Gaussian source is to consider the full width at half-maximum (FWHM) phase space dimensions of the source in each transverse direction, and divide by the selected X-ray wavelength λ. One thus arrives at a measure of the number of spatially coherent ‘modes’ in each transverse direction, and one can then see how many modes need to be rejected in order to achieve a focus limited by diffraction of the nanofocusing optic rather than by the dimensions of the photon source. In Table 1 ▶, we show an example calculation of this sort for the Advanced Photon Source (APS) at Argonne National Laboratory in the USA, where we use the present-day source parameters as well as an example set of parameters estimated for a possible multi-bend achromat (MBA) upgrade of the storage ring lattice. As can be seen, the gain offered by the diffraction-limited storage ring upgrade is significant: one goes from having to ‘throw away’ 437 modes today at 10 keV to only removing 4.7 modes with the upgrade. The MBA lattice upgrade of the APS is also planned to include a doubling of the storage ring current, and improved undulators, so the gains in coherent flux will be even higher.

**Figure 4:**
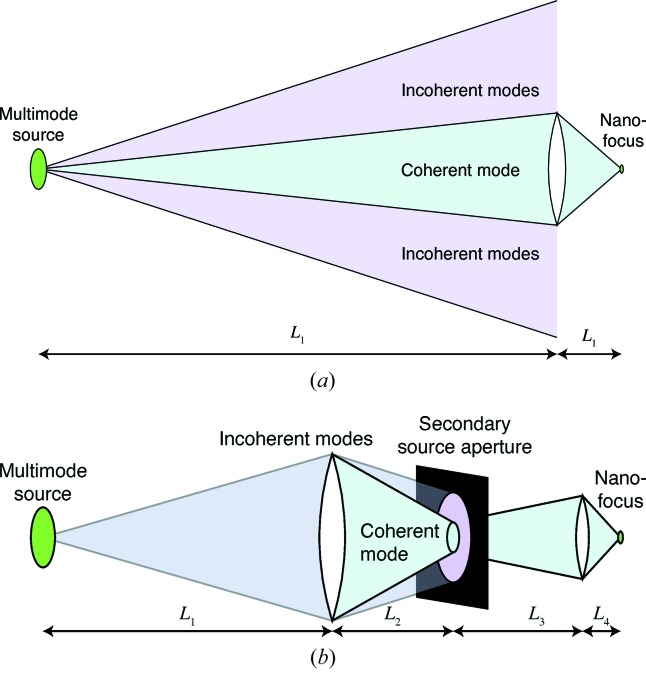
Schematic of one-stage (*a*) and two-stage (*b*) beamline optical layouts for selecting a single coherent mode for nanofocusing experiments. As described in §3, one-stage schemes offer minimal flux loss due to imperfect beamline optics, while two-stage schemes offer shorter beamlines and greater control over flux *versus* resolution trade-offs.

When the radiation source has many modes that must be thrown away in order to achieve a diffraction-limited focus, one can be very flexible with the optical design of a beamline; for example, one does not have to be concerned with the tilting of phase space ellipses with propagation distance (Hastings, 1977 ▶; Pianetta & Lindau, 1978 ▶; Smilgies, 2008 ▶; Ferrero *et al.*, 2008 ▶; Huang *et al.*, 2010 ▶) which can otherwise lead to a loss of useful flux and an undesired correlation of angle with position in the illumination arriving at apertures or beamline optics. With a diffraction-limited storage ring, one must be much more careful to preserve the acceptance and coherence of the selected mode; this involves both increased precision of the optics as described elsewhere in this special issue, and also proper optical design. In Fig. 4 ▶, we show two alternative schemes for selecting a single coherent mode from a nearly diffraction-limited source:

(i) One option (Fig. 4*a* ▶) is to place the nanofocusing optic at some distance from the source, so that it accepts only a single coherent mode and demagnifies the source directly. Because no beamline focusing optics or apertures are used between the source and the nanofocusing optic, one does not lose flux or degrade the coherence of the central mode due to imperfections in beamline optics. However, one must then choose the diameter of the nanofocusing optic based on properties of the source; this sets conditions on the optic that may not be optimal due to other considerations, such as minimum focal length due to working distance constrains, or maximum diameter due to thickness limits in multilayer Laue lenses (Maser *et al.*, 2004 ▶), or field diameter limits in electron beam lithography of Fresnel zone plates. One may also require different desired optic diameters in the horizontal and vertical directions, complicating the use of circular zone plates or refractive lenses. This approach tends to lead to long beamlines, with attendant conventional construction costs. For example, to demagnify a 40 µm source (the horizontal source size expected for the MBA lattice upgrade of the APS) to a 20 nm spot while using a nanofocusing optic with a convenient focal length of 0.1 m, one would need to have the distance 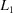 in Fig. 4(*a*) ▶ be about 200 m. Finally, oscillations in source position will lead to oscillations in the probe position or, equivalently, pixel position errors in scanning microscopy; oscillations in the source angle will lead to oscillations in focused beam intensity only to the degree which the source has one or a few modes.

(ii) Another option (Fig. 4*b* ▶) is to use beamline optics to image the source onto a secondary source aperture, which can be adjusted to pass one or several coherent modes. This allows the experimenter to make a flux-*versus*-resolution trade-off if the source has multiple coherence modes, and it also allows one to reject beamline-optics-caused degradations of the coherence of a single mode (spatial filters in visible-light laser laboratories work on the same principle by placing a pinhole at a lens focus) at the cost of a further loss of flux. Oscillations in either source position or angle would lead to oscillations in the focused beam intensity, which can in principle be corrected using a mostly transparent beam flux monitor (such as a thin diamond film) after the secondary source aperture. One can also adjust the secondary source aperture’s diameter, and distance 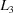 to the nanofocusing optic (Fig. 4*b* ▶), so as to accommodate a desired nanofocusing optic diameter. By using two-stage demagnification, one can design a shorter beamline with lower conventional construction costs; for example, if one chose 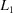 = 30 m due to the distance at which a first optic can be placed after an accelerator shield wall and 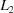 = 3 m, one can demagnify a 40 µm source to 4 µm at the point of the secondary source aperture, and then if 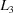 = 20 m to work with a nanofocusing optic focal length of 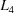 = 0.1 m, one will have demagnified a 40 µm source to a 20 nm focus in a total beamline length of only 53.1 m. One of the engineering challenges in this approach is to make high-quality controllable apertures of just a few micrometers in width and with the ability to handle high power densities.

Because the on-axis spectral linewidth of undulator radiation is affected by angular divergence of the source, diffraction-limited storage rings will provide improved spectral purity which can also be exploited to yield further gains in the flux of a nanofocused beam. Rather than use a crystal monochromator with a bandpass of the order of 0.1–1 eV, one could use a multilayer-coated nanofocusing mirror (or a deflecting mirror or a double multilayer monochromator) to select the entire spectral width of the undulator’s harmonic output. Smaller-diameter Fresnel zone plates have only 100–200 zones, and again they could use the entire spectral output of the source as could a conventional non-multilayer-coated Kirkpatrick–Baez reflective nanofocusing system.

**Figure 5:**
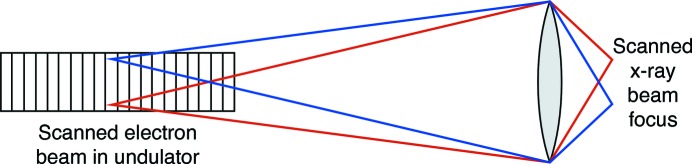
Fast scanning by moving the radiation source rather than the optic or the specimen. As will be seen in Table 2 ▶, the pixel transit time of X-ray fluorescence nanoprobes could potentially go to around 10 µs. This would lead to high velocities and acceleration/deceleration cycles on scanned optics or specimens. An alternative in the case of single optic beamline designs (Fig. 4*a* ▶) and true single-coherent-mode sources could be to scan the source in both position (to move the nanofocus beam point) and angle (so that the beam illuminates the nanofocusing optic as the source position changes). The range in electron beam position scanning would be large; for example, the demagnification of 350 required to image a 7 µm source to a 20 nm focus would require the source to be scanned over a 3.5 mm range in order to produce a 10 µm scanned image field. This would be challenging for electron beam orbit feedback systems to maintain stability, and it may therefore be unrealistic; however, fast scanning of specimens or optics is also challenging.

As will be seen in Table 2 below, the advances of diffraction-limited storage rings have the potential to be coupled with other potential gains in nanoprobe instrumentation to deliver pixel transit times in the microsecond range. The rapid scan speeds involved (along with the acceleration and deceleration at the start and end of scan lines) are not easy to contemplate for mechanical scanning of the nanofocusing optic or the specimen. (Most scanning X-ray microscopes scan the specimen, because optic scanning has the potential complication of producing optic position-dependent flux and wavefield variations if there is any unevenness in the optic’s illumination; this becomes particularly important for ptychography, where fluctuations in the probe become difficult to disentangle from structure in the object.) With the single optic beamline scheme of Fig. 4(*a*) ▶, one could consider a scheme that pleases the scanning microscope designer and horrifies the accelerator physicist: scanning of the source in position and angle, as shown in Fig. 5 ▶. This would work best in the vertical direction, where diffraction-limited storage rings will offer a source phase space of about 1λ. It would require a significant excursion of the electron beam within the undulator (up to a few millimeters), and place stringent demands on beam position monitors and orbit feedback systems. It would also work for only a limited field of view, though in principle one could ‘tile’ together multiple small scan areas with sample translation used to go to new ‘tile’ centers. A crazy scheme, perhaps; but the mass of the electrons in a bunch in the storage ring is astronomically smaller than the mass of a scanned specimen and nanopositioning stage.

It is worth commenting on the role the spatial resolution of the nanofocusing optic plays in the capture of coherent flux from the source. One might think that a higher resolution optic would require more stringent coherence from the source, and thus lower the flux in the focused beam. However, the phase space acceptance [equation (1)] of an optic free from aberrations is 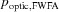 = 2.44λ, independent of the numerical aperture and thus theoretical resolution of the optic. Consider the case of an optic with an adjustable aperture; for a low aperture one can place the optic closer to the source so that it is filled with a coherent mode, and the source is only weakly demagnified. As the aperture is increased, the optic should be moved farther from the source in order to still be filled with a coherent mode of the same angular extent from a given source size, and the source will be more strongly demagnified to a smaller spot. In both cases the optic should be illuminated only with a coherent mode, so that it employs the same coherent flux from the source. We qualify this statement with the following comments:

(i) One can always gain additional flux by accepting additional modes from the source, but this will lead directly to a larger focused beam spot and a corresponding decrease in spatial resolution (Winn *et al.*, 2000 ▶).

(ii) If one uses a dispersive optic such as a Fresnel zone plate, multilayer Laue lens or compound refractive lens, the higher-resolution optic will have larger chromatic disperson (*e.g.* zone plates will have more zones) for a given focal length, and this will require smaller bandpass from the source. Even so, the bandpass of the optic may still be comparable with the bandwidth of the undulator source as noted in the next section, so in fact this may not have a large impact on potentially available focused flux.

(iii) Higher resolution optics often have shorter focal lengths. (For example, with Fresnel zone plates one may choose to limit the diameter of the optic due to limitations on the field size in high-accuracy electron beam lithography systems, or on the number of zones in the optic and thus the required illumination monochromaticity; either choice leads to a shorter focal length as the resolution is improved.) The reduced working distance leads to challenges in microscope design, but, given that soft X-ray scanning microscopes often work with optics with a focal length of only a few millimeters, these challenges for hard X-ray fluorescence nanoprobes may not be insurmountable.

(iv) For Fresnel zone plates, higher resolution optics correspond to narrower zones, which often means decreased thickness (due to nanofabrication aspect ratio limits) and thus reduced focusing efficiency. In the next section we discuss developments that have the potential for removing this limitation to yield improved nanofocusing optics.

(v) On the specimen side, higher resolution features tend also to be thinner and thus have decreasing contrast, so the signal required for feature detection tends to increase with the fourth power of decreases in feature size (Howells *et al.*, 2009 ▶). However, in fluorescence if one has a fixed areal concentration of atoms of a particular species, reducing the beam spot size while maintaining the beam flux leads to the same fluorescence signal: a smaller number of atoms are nonetheless ‘hit’ by the same number of incident photons, thus producing the same number of fluorescent photons.

For these reasons, one can expect future improvements in spatial resolution in X-ray fluorescence nanotomography while simultaneously decreasing imaging time.

### 3.1. General consequences of DLSR upgrades: flux and sensitivity estimates   

In order to discuss the opportunities for X-ray fluorescence tomography it is useful to have some definition of the anticipated measurement parameters. Here we use the parameters obtained from an operating cryo X-ray fluorescence nano­probe, the Bionanoprobe at the APS (Chen *et al.*, 2014 ▶), as a starting point for this estimate. This instrument operates with a source brightness of about 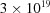
 photons s^−1^ mm^−2^ mrad^−2^ (0.1% bandwidth)^−1^ at 10 keV, and a beamline optical scheme that resembles Fig. 4(*b*) ▶ except with source apertures located at 27 m from the source, while the microscope is about 67 m from the source. The beamline uses a double-crystal monochromator with an energy bandpass of about 1.5 eV. Using a zone plate with an efficiency of about 8% at 10 keV, a flux of about 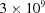
 photons s^−1^ is delivered into a spot with a theoretical Rayleigh resolution of 85 nm, a resolution consistent with that observed in images of microfabricated test patterns. The instrument uses a silicon drift diode fluorescence detector with a solid angle coverage of about 0.65 sr.

When this instrument is used to scan a frozen hydrated algae of thickness a few micrometers, the total signal rate in the fluorescence detector is about 30000 counts s^−1^. However, this count rate is dominated by both elastic and Compton scattered photons. The fluorescence signals for biologically significant elements such as K, Mn, Fe and Zn are more typically in the range 300–2000 counts s^−1^. Many of the fluorescence images have been taken using ‘step scans’ where the sample is moved to a new point in the raster scan, data are collected for a dwell time (typically 1 s), and the sample is then moved to the next position in the scan field. The microscope is also able to operate in a so-called ‘fly scan’ mode where the sample is continuously moved through a scan line, and the detector signal is recorded over fixed distance increments. Fly scans with per pixel transit times of as low as 5 ms have been employed thus far with this instrument (and pixel transit times down to 0.050 ms have been used for fly scans at the Australian Synchrotron).

We now scale the performance of this instrument to what one could hope for with the APS MBA lattice upgrade as an example of a diffraction-limited storage ring, and upgrades in beamline optics and microscope instrumentation. From Table 1 ▶ we see that the phase space product 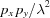 of the source decreases by 77, while the beam current will be doubled so that the overall gain in brightness is about 150. This is the single most dramatic factor that can improve the capabilities of X-ray fluorescence nanoprobes for microscopy and tomography, but one can also consider additional gains:

(i) *Increased spectral bandpass.* The spectral linewidth of on-axis radiation from an undulator is approximately equal to 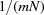, where *N* is the number of magnetic periods of the undulator and *m* is the radiation harmonic one works with. Undulators on diffraction-limited storage rings might have ∼200 periods, and depending on electron energy will use harmonics 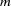 = 1, 3, 5 for highest brightness at 10 keV, so the spectral linewidth will be about 10–50 eV. In contrast, silicon 〈111〉 monochromators have a natural bandpass of only about 1.5 eV at these energies, so that they effectively throw away a factor of 5–25 in useable photon flux. If single-coating Kirkpatrick–Baez mirrors were to be used for nanofocusing, one could in principle work without a monochromator, while if one were to use Fresnel zone plates (which rarely have more than 1000 zones) one would require no greater than 10 eV bandpass in the illumination. As an example of the gain in focused flux that one might be able to obtain by using a multilayer-based monochromator rather than a crystal monochromator, we list a potential flux gain of a factor of ten in Table 2 ▶.

(ii) *Improved nanofocusing optics.* The Bionanoprobe uses two ‘stacked’ Fresnel zone plates (Shastri *et al.*, 2001 ▶) to add thickness in the X-ray beam direction and thus efficiency (Kirz, 1974 ▶). However, there is considerable room for further gain in the efficiency of nanofocusing optics. High-aspect-ratio fabrication schemes such as zone doubling (Jefimovs *et al.*, 2007 ▶) are increasing the efficiency of single Fresnel zone plates with zone widths of 20 nm or smaller. The stacking of multiple zone plates has been proposed to move from established near-field schemes, where identical zone plates are within a depth of focus of each other, to arrangements where the separation distances are larger and the diameters of multiple individual zone plates are adjusted to a common focus (Vila-Comamala *et al.*, 2013 ▶). For applications where wide-band energy tuning is not required, multilayer Laue lenses (Maser *et al.*, 2004 ▶; Yan *et al.*, 2014 ▶) are now achieving focal spots near 10 nm with an efficiency of 15% in a scheme where further gains can be anticipated. For wide-band applications, Kirkpatrick–Baez reflecting optics have achieved a 7 nm focus in one-dimensional examples (Mimura *et al.*, 2010 ▶), and systems with two-dimensional foci below 50 nm are now commercially available. Altogether the prospects for increased efficiency of nano­focusing optics are quite favorable, and it is not unreasonable to expect a gain of a factor of three beyond what is available in the Bionanoprobe today.

(iii) *Improved detector solid angle.* X-ray fluorescence is emitted into a solid angle of 4π steradians, so the acceptance angle of the detector directly affects the measured signal. In electron microscopy, there have been demonstrations of detectors with a solid angle of collection of π steradians though with limitations on the maximum count rate (Zaluzec, 2009 ▶); in §4.5 we describe the Maia detector and the increase of a factor of 1.8 in collection angle it provides compared with what is used in the Bionanoprobe today.

It may be difficult to realise the indicated values of these gains either separately or especially collectively, but it it is still worthwhile considering the possibilities. As Table 2 ▶ shows, the aggregate effect of these potential improvements is staggering: one can contemplate detecting 100 photons in a fluorescence line from a typical elemental concentration in a cell with pixel transit times of only 12 µs, even as the spatial resolution is improved.

## 4. Fluorescence nanotomography: challenges and opportunities   

It is clear from the flux and pixel transit times above that X-ray fluorescence nanotomography experiments with diffraction-limited storage rings will be very different than experiments today. This creates opportunities for new approaches and capabilities. We will consider the case of 15 nm voxel resolution tomography with a pixel transit time of 12 µs based on Table 2 ▶.

### 4.1. Depth of field and sample size limits   

Basic tomography assumes that features throughout the depth of the object are recorded faithfully in each projection image. In a microscope, this is true only if the object lies within the depth of focus of the microscope. The Rayleigh resolution 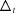 of a lens is given by 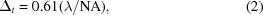
where NA is the numerical aperture of the lens and λ is the radiation wavelength. The depth resolution (Born & Wolf, 1999 ▶) 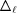 is given by 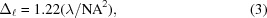
and the depth of focus is twice the depth resolution (Wang *et al.*, 2000 ▶). If an object of depth and width 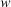 = 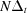 is to be imaged in the pure projection approximation so that the Radon transform applies, the width cannot exceed 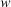 = 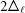 so that one arrives at a limit on the number of voxels *N* on a side of 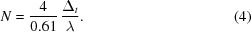
For 15 nm resolution at 10 keV, one arrives at 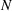 = 794 or an object dimension of 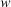 = 11.9 µm. Assuming the Crowther criterion (Crowther *et al.*, 1970*a* ▶,*b* ▶) for tomography which works out to collecting projections from 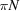 rotation angles, the total number of data points to be collected is 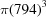 or 1.57 GigaSamples, highlighting the challenges of data processing which will be described in §5. To scan larger objects, one can use higher photon energies (thus decreasing λ), or acquire a through-focus set of projections (in which case the incoherent nature of the fluorescence imaging process simplifies data processing), or turn to diffraction tomography reconstruction approaches.

### 4.2. Alternative scanning modes   

If we scan a 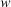 = 11.9 µm-wide object at 15 nm pixel size, with 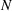 = 794 pixels per scan line at 12 µs pixel transit time, each scan line would take 9.5 ms to acquire and (neglecting acceleration, deceleration and flyback times) the sample would be shaken at 105 Hz. [The scan velocity would be 1250 µm s^−1^, and the total data acquisition time with no flyback or ‘overhead’ time losses would be about 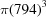 × 12 µs or about 5.2 h.] Given the delicacy of many biological specimens, and the complications of imaging them at cryogenic temperatures (§4.3), there are sufficient reasons to consider alternative means for scanned image acquisition. One approach is to scan the X-ray beam rather than the sample, either by scanning the electron beam in the storage ring as illustrated in Fig. 5 ▶ or by scanning the focusing optics. This latter approach is not without complications: it seems impractical when considering high-mass systems such as Kirkpatrick–Baez mirrors, and one would need to account for vibration when working with multiple stacked zone plates (Shastri *et al.*, 2001 ▶; Vila-Comamala *et al.*, 2013 ▶). In addition, the combination of fluorescence with differential phase contrast (§4.6) or ptychography (§4.7) might be compromised as any changes in illumination of the nanofocusing optic would be difficult to disentangle from perceived structure in the specimen.

One alternative approach is to consider a change in the order of scan axes. Up until now, most fluorescence tomography data have been collected *via* a series of 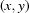 raster scans or projections at each of a set of rotational angles θ; that is, in the order 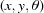 (see Fig. 2 ▶ for a definition of coordinates). This scan ordering has the disadvantages that no complete sinogram is measured until the last projection has been acquired, and that positional drifts during the projection series are, if uncorrected, fatal to the entire dataset. One could instead consider an approach where the sample is instead scanned through θ first at each of a set of 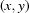 positions. A rotation axis could spin continuously with no further acceleration, for part or all of the entire measurement (this would of course be complicated if the sample had to be cooled through heat-conducting wires or cold gas tubes). The *x* position could be stepped incrementally once per rotation, or slewed continuously at the appropriate velocity, with minimal missing wedge in the tomogram or loss of resolution (Kosior *et al.*, 2012 ▶). By measuring in the scan order 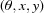 one effectively acquires a series of single-slice tomograms (like the optical slice shown in Fig. 3 ▶), with one complete slice ready for reconstruction every 24 s if 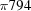 = 2494 angular steps were used. In addition to the preferred mechanics of this scan ordering, significant benefits include the ability to test data quality while the scan is being acquired, insurance against incomplete scans, and a shorter time over which one must demand no specimen drift. It should be noted that this fast-angular mode requires the specimen to be mechanically aligned to the rotation center, as characterization of the (non-centered) specimen locus and dynamic correction for specimen center may not be feasible at such high rotational speeds.

### 4.3. Specimen preservation: radiation dose and cryogenics   

Because X-ray fluorescence is an indirect signal that follows X-ray absorption in an atom from the element being imaged (which might be a trace element), it involves a high radiation dose. In the case of illuminating a sample comprised mainly of protein with 10 keV X rays at a flux of 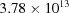
 photons s^−1^ into 15 nm pixels at 12 µs pixel transit time, we estimate that the specimen will receive a radiation dose for a two-dimensional image of 1300 MegaGray (MGy). This dose is about 30 times higher than the 43 MGy point at which the intensity of diffraction spots from cryo-cooled protein crystals is reduced by half (Owen *et al.*, 2006 ▶), so the atomic scale structural arrangement of organic molecules will be severely compromised. However, for fluorescence analysis we have a different criterion: are the fluorescent atoms still there in the same number within the size of the beam spot? In experiments aimed at single atom detection using electron-energy-loss spectroscopy in transmission electron microscopy, Leapman (2003 ▶) has estimated that metal atoms were preserved in dehydrated specimens at electron exposures of 10^9^ e^−1^ nm^−2^, which we estimate (Jacobsen *et al.*, 1998 ▶) to correspond to a dose of about 3 × 10^7^ MGy. Radiation dose effects are always sample-dependent and should be checked as part of careful experimental protocol, but these considerations suggest that the fluorescence signal from dehydrated specimens is unlikely to be affected at the doses involved in experiments with diffraction-limited storage rings.

Dehydrated biological specimens are not always faithful to nature, and in a perfect world one would image trace-metal content in living cells. Unfortunately, wet functional myofibrils are deactivated at radiation doses of about 0.01 MGy (Bennett *et al.*, 1993 ▶), initially living fibroblast cells show immediate degradation at doses of about 0.1 MGy (Gilbert & Pine, 1992 ▶; Kirz *et al.*, 1995 ▶), and X-ray fluorescence microscopy studies of vanadium in room-temperature hydrated ascidian blood cells showed a loss in signal at doses of 0.1 MGy (Fayard *et al.*, 2009 ▶). Room-temperature hydrated samples that have been chemically fixed show greater variation in dose response, with chromosomes showing mass loss and shrinkage at doses of about 100 MGy (Williams *et al.*, 1993 ▶) while other samples such as malaria-infected erythrocytes showing higher dose tolerance (Kirz *et al.*, 1995 ▶). Chemical fixation is sometimes used to preserve cellular structure against the loss of membrane integrity that can occur during dehydration, but fixation protocols can themselves lead to alterations in trace-element content (Chwiej *et al.*, 2005 ▶; Matsuyama *et al.*, 2010 ▶; Hackett *et al.*, 2011 ▶). Rapid freezing followed by freeze-drying provides a less chemically disruptive approach, but cannot preserve three-dimensional specimen ultrastructure at the highest fidelity.

The ‘gold standard’ of sample preparation is to work with rapidly frozen samples imaged in a frozen hydrated state at temperatures below the vitreous ice recrystalization temperature of about 135 K. This approach has been developed with great success in biological electron microscopy (Steinbrecht & Zierold, 1987 ▶; Dubochet *et al.*, 1988 ▶), showing excellent preservation of cellular structure and content. This approach has been carried over to soft X-ray microscopy for full-field imaging (Schneider, 1998 ▶) and tomography (Schneider *et al.*, 2002 ▶, 2010 ▶; Larabell & Le Gros, 2004 ▶), scanning microscopy (Maser *et al.*, 2000 ▶) including the first tomograms of whole frozen hydrated mammalian cells (Wang *et al.*, 2000 ▶), and coherent diffraction imaging (Beetz *et al.*, 2005 ▶; Huang *et al.*, 2011 ▶). Since the first demonstration (Maser *et al.*, 2000 ▶), there have been no other cryo scanning X-ray microscopes in operation until more recent X-ray fluorescence microscope demonstrations using conductive cooling of specimens in a vacuum environment (Matsuyama *et al.*, 2010 ▶; Dučić *et al.*, 2011 ▶; Chen *et al.*, 2014 ▶). While cryo X-ray fluorescence tomography is only now beginning to be demonstrated (Yuan *et al.*, 2013 ▶), we feel that cryo imaging represents the future for biological studies, especially with the speed and sample size capabilities that diffraction-limited storage rings will offer. Dedicated facilities for cryo sample preparation, with plunge and high-pressure freezers, cryo ultramicrotomes, and cryo light microscopes, with the commensurate expertise, will soon be a standard requirement for X-ray fluorescence nanotomography.

Cryogenic temperatures do not make specimens impervious to radiation dose in X-ray microscopy. As noted above, some protein crystals show a loss of half of their diffraction signal at radiation doses of 43 MGy (Owen *et al.*, 2006 ▶), and extrapolations of a large set of data have been used to suggest that a resolution of about 10 nm can be obtained in X-ray microscopy studies of biological specimens (Howells *et al.*, 2009 ▶). Soft X-ray absorption spectroscopy shows that there is significant bond disruption in organic materials at doses of about 10 MGy, yet at the same time that there is very little mass loss (Beetz & Jacobsen, 2003 ▶). This is consistent with the *absence* of observed radiation damage effects in 30–100 nm resolution cryo X-ray microscopy of cells at doses up to about 10^4^ MGy (Schneider, 1998 ▶; Maser *et al.*, 2000 ▶). Coupled with the lack of observation of elemental signal loss at even higher doses in room-temperature electron-energy-loss imaging experiments (Leapman, 2003 ▶), we can be hopeful that cryo X-ray fluorescence nanotomography will yield faithful measurements of elemental signal content even when diffraction-limited storage rings are used.

### 4.4. Opportunity: dose fractionation   

Dose fractionation is the idea that ‘a three-dimensional reconstruction requires the same integral dose as a conventional two-dimensional micrograph provided that the level of significance and the resolution are identical. The necessary dose *D* for one of the *K* projections in a reconstruction series is, therefore, the integral dose divided by *K*’ (Hegerl & Hoppe, 1976 ▶). Consider a particular voxel in a volumetric reconstruction: to see that the material in this voxel is different than what is in the adjoining voxel, one needs to have determined the number of photons arising (in our case) from each voxel. In a two-dimensional image, these photons will all have been collected from one angle, yielding no depth information. In tomography, the information from a voxel will be distributed among specific positions in each of the individual projections, but that information is reorganized into voxels (or, from the point of view of one particular voxel, collected from the set of projections) during the act of tomographic reconstruction. It matters not at all the direction from which different photons were collected into a voxel; what matters is that the total number of photons needed to recognize its contents were obtained. Dose fractionation was originally a controversial concept (Hoppe & Hegerl, 1981 ▶), but in fact it has emerged as a necessary condition for the success of modern single-particle electron microscopy methods (Nogales & Grigorieff, 2001 ▶). The validity of dose fractionation has also been demonstrated carefully by McEwen *et al.* *via* the controlled circumstances of simulations; they stated ‘the simulations verify the basic conclusions of the [dose fractionation] theorem and extend its validity to the experimentally more realistic conditions of high absorption, signal-dependent noise, varying specimen contrast and missing angular range’ (McEwen *et al.*, 1995 ▶). We therefore see that dose fractionation should allow us to reduce the dose and increase the speed of data collection in X-ray fluorescence tomography; however, while there has been some speculation of its potential (de Jonge & Vogt, 2010 ▶; Lombi *et al.*, 2011*b* ▶), we are unaware of any work in which dose fractionation has been employed in a systematic and well characterized way in X-ray fluorescence tomography.

Dose fractionation appears as a consequence of the reconstruction process, and so in a sense one does not need to do anything to benefit from it. In one fluorescence nano­tomography example (de Jonge *et al.*, 2010 ▶), it was observed that the statistical level of the reprojection from the entirety of the reconstructed data was higher than that of the relevant individual projection: this was due to dose fractionation. However, it is likely that extreme fractionation (the acquisition of very many projections of very low individual statistical level) will require reconstruction approaches designed explicitly to preserve this type of information, as well as correlative imaging methods to aid in projection alignment (§4.6). Finally, we note that the estimation in §4.3 of radiation dose for a two-dimensional image would apply to a three-dimensional tomographic dataset if dose fractionation were to be used, and that the scanning speeds discussed in §4.2 would need to increase if one were to take advantage of dose fractionation and acquire fewer photons per projection.

### 4.5. Energy-resolving detectors   

The potential gains outlined in Table 2 ▶ are only realisable if the signals can be detected. Today most X-ray fluorescence microscopes utilize four-element silicon drift detectors for energy-dispersive spectroscopy of the emitted fluorescence signal; these detectors offer simplicity of operation, good energy resolution (typically about 130 eV at 5.6 keV), and good efficiency for detecting photons in the 2–15 keV energy range. The fastest electronics today allow single elements to handle a total detected flux (including elastic and Compton scattered photons) of about 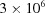
 photons s^−1^ (with some spectral degradation and significant pileup) with accumulation times (pixel transit times) as small as about 1 ms; this puts multielement detectors within reach of meeting the 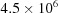
 photons s^−1^ count rate and 0.67 ms pixel transit time estimated in Table 2 ▶ for diffraction-limited storage rings. With additional improvements in spectral bandpass and nanofocusing optic efficiency, today’s commercial detectors are *not* up to the required performance, so one must think of alternative fluorescence detection systems.

Because the undulator-produced X-ray beam at modern synchrotron light sources is horizontally polarized, elastic scattering is minimized when the fluorescence detector is located at a horizontal angle of 90° relative to the incident beam. With limited count-rate capabilities of existing detectors, this has been considered to be one of the keys to realising an extremely low background signal in X-ray fluorescence microscopy, optimizing the sensitivity with minimum radiation dose (Kirz, 1980*a* ▶; Sparks, 1980 ▶). However, such detectors have traditionally occupied less than 0.1 sr of the overall 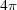 solid angle (0.8%). This is being changed by the use of four-element detectors that collect from a solid angle extending well beyond this scatter minimum, so it is worthwhile considering the trade-off between minimizing the scattered signal and maximizing the collection of fluorescence photons. A recent study (Ryan *et al.*, 2014 ▶) investigating the influence of detection angle from 90 to 180° (backscatter) geometries showed that the advantage of 90° detection diminishes as large collection solid-angle is pursued. Greatly increased solid angle can compensate for increased background, even in the backscatter geometry, thereby increasing counting statistics essential for quality imaging and ultimate elemental sensitivity. This has enabled the backscatter geometry to be used to allow large 2D scanning ranges and extended sample areas while maintaining large collection solid angle. Even so, for the small samples one is likely to study in a nanoprobe, the 90° geometry remains the preferred choice.

One must still face the challenge of the very high count rates that Table 2 ▶ points to with improvements beyond the realisation of diffraction-limited storage rings. The best path for reaching higher count rates is to divide the detector into a larger number of smaller elements. This is the approach taken by the Maia detector, in which the active area is divided up into 384 detectors to provide total count rates of 4 to 12 million counts per second (Kirkham *et al.*, 2010 ▶; Ryan *et al.*, 2014 ▶). An additional innovation has been the switch to an event-based acquisition mode, with the Maia detector real-time processor tracking specimen position encoders and determining pixel-boundary crossing so that detected photons are tagged with their pixel position. When imaging mineral specimens where metal contents are far above the trace levels seen in biological specimens, the Maia detector has already been used with pixel transit times of between 50 µs (Ryan *et al.*, 2010*b* ▶) and 500 µs (Ryan *et al.*, 2013 ▶; Dyl *et al.*, 2014 ▶) for the acquisition of fluorescence maps with up to 100 million pixels at high statistical level and part-per-million sensitivity. (The segmentation or ‘pixelation’ of the Maia detector also allows one to make analysis adjustments after-the-fact of the scan by excluding the events from the outer diameter detector elements, so as to explore the scatter *versus* fluorescence photon collection trade-offs that go with increasing collection solid angle.) For studies of lower elemental concentrations in biological and environmental specimens (Lombi *et al.*, 2011*a* ▶,*b* ▶; McColl *et al.*, 2012 ▶; James *et al.*, 2013*a* ▶), the 1.2 sr solid angle of the Maia detector used in the backscattering geometry (Ryan *et al.*, 2010*a* ▶) collects significantly more photons than the detector used today in the Bionanoprobe. This gain in collection solid angle is the basis for the estimated gain in the bottom row of Table 2 ▶ though with some samples it comes at the cost of a 50% increase in the scatter background signal (not reflected in Table 2 ▶).

While the Maia detector represents a development towards realising the potential that diffraction-limited storage rings and other improvements might offer, neither it nor any other detector now available is ready to handle the count rates one could hope for with all the possible gains listed in Table 2 ▶. This points to the clear need for ongoing improvements in detector technology to match the worldwide investment in diffraction-limited storage rings.

### 4.6. Correlative signals and complementary contrast   

Today’s X-ray fluorescence microscopes have exquisite sensitivity for detecting biologically important metals such as calcium, manganese and iron. However, they show less sensitivity for the detection of the light elements (H, C, N and O) that comprise most of the mass and structure of biological specimens. As shown in Fig. 1 ▶, the fluorescence yields for these elements are quite low, and moreover most fluorescent photons from these elements are re-absorbed within the cell or tissue. While there are notable exceptions of systems designed to successfully detect fluorescence from elements as light as carbon (Kaulich *et al.*, 2009 ▶), in general one must use other means to image the main mass and structure of cells and tissues in X-ray fluorescence microscopes.

Phase contrast provides such a means (Schmahl & Rudolph, 1987 ▶). At 10 keV, the phase shifting part δ of the X-ray refractive index *n* = 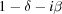 for protein is about 560 times larger than the absorptive part β (Henke *et al.*, 1993 ▶), so that phase contrast allows for easy viewing of thin biological specimens that are almost invisible in absorption contrast (Hornberger *et al.*, 2008 ▶). Differential phase contrast is easily realised in hard X-ray fluorescence microscopes by using various segmented detector schemes (Morrison, 1992 ▶; Kaulich *et al.*, 2002 ▶; Feser *et al.*, 2006 ▶; Hornberger *et al.*, 2008 ▶), and various analysis schemes can reconstruct the phase contrast image from these signals (Hornberger *et al.*, 2007 ▶; de Jonge *et al.*, 2008 ▶). Since differential phase contrast works well with as few as four segments to the transmission detector (Hornberger *et al.*, 2007 ▶), the additional data storage is negligible so that differential phase contrast images can be recorded routinely with all fluorescence maps. Data recording can be simplified further if one uses the Zernike method (Holzner *et al.*, 2010 ▶) to record the phase contrast image using a single detector segment. One can also use propagation-based phase contrast images recorded subsequently to X-ray fluorescence maps using the same instrument (Bleuet *et al.*, 2009 ▶). As noted in §2, the phase contrast image can be used to estimate sample mass and thus obtain quantitative mass concentration from fluorescence signals (Holzner *et al.*, 2010 ▶; Kosior *et al.*, 2012 ▶), along with providing a view of specimen ultrastructure so that elemental content can be put into its functional context.

Phase contrast can manifest itself in additional ways. As variations are imposed upon a wavefield, part of the energy is redirected into directions other than the forward direction. If one can separate the forward-directed beam from the scattered beam, one can obtain a dark-field image that highlights phase (and absorption) variations in the object. This can be done in scanning microscopes with a simple beamstop on a transmission detector (Morrison, 1992 ▶; Chapman *et al.*, 1996 ▶), or through the use of a pixelated area detector as has been demonstrated with soft (Chapman *et al.*, 1995 ▶; Morrison *et al.*, 2006 ▶; Gianoncelli *et al.*, 2006 ▶) and hard (Thibault *et al.*, 2009 ▶; Menzel *et al.*, 2010 ▶) X-ray scanning microscopy. The integration of fast detectors into advanced control systems allows for easy combination of these imaging modes (Medjoubi *et al.*, 2013 ▶), and of course these developments also lead one into ptychography as is discussed in the following section.

Two further contrast methods have recently been exploited in fluorescence imaging and tomography due to a re-evaluation of the optimal fluorescence detector geometry: Rayleigh and Compton scatter contrast. The backscatter geometry employed by the Maia detector system results in strong Compton and Rayleigh scattering signals, and these can easily represent some 90% of photon events from biological samples. In the absence of crystallinity and diffraction these signals are both linear and strong, and have been used for both alignment and ultrastructural visualization for fast fluorescence tomography of metal uptake in hydrated plant roots (Lombi *et al.*, 2011*a* ▶), and of the ultrastructural matrix of a whole *C. elegans* (McColl *et al.*, 2012 ▶). High-definition Compton tomography has been used to provide clear ultrastructural visualization in whole rice grains, as shown in Fig. 3 ▶ (Carey *et al.*, 2011 ▶).

When combining signals from multiple contrast mechanisms, one must worry about alignment of different images if they were not taken in simultaneous measurements. This can add additional complications to the projection alignment problem discussed in §5.1.

We have limited our discussion to the detection of the presence of specific elements through X-ray fluorescence. Of course one can also obtain information about the chemical state of elements by selectively exciting transitions of core-level electrons into partially occupied low-binding-energy electronic states which are affected by the chemical bonds an atom is participating in. This gives rise to near-edge X-ray absorption fine structure (NEXAFS) or X-ray absorption near-edge structure (XANES) contrast. This is widely used for chemical state imaging in soft X-ray microscopy (Ade *et al.*, 1992 ▶; De Stasio *et al.*, 1993 ▶; Jacobsen *et al.*, 2000 ▶), and for speciation studies in hard X-ray microprobes (Sutton *et al.*, 1995 ▶; Schroer *et al.*, 2003 ▶; Gräfe *et al.*, 2014 ▶) including for tomography applications (Golosio *et al.*, 2004 ▶). However, radiation damage can affect studies of chemical speciation even with samples studied under cryogenic conditions (Beetz & Jacobsen, 2003 ▶; Corbett *et al.*, 2007 ▶; George *et al.*, 2012 ▶), so uncertainties remain regarding the potential utilization of XANES for chemical speciation studies in X-ray fluorescence nanotomography.

### 4.7. Ptychography   

X-ray ptychography (Faulkner & Rodenburg, 2004 ▶; Rodenburg & Faulkner, 2004 ▶; Thibault *et al.*, 2008 ▶) is a variant of coherent diffraction imaging (CDI) or X-ray diffraction microscopy (Miao *et al.*, 1998 ▶) that uses overlapping measurements of coherent diffraction patterns to recover both the specimen and probe functions at resolutions that are, in principle, only wavelength limited. Ptychography requires the recording of diffraction patterns with a pixelated area detector, and this detector must have an image frame rate that matches the desired illumination spot scan rate. In most cases ptychography is carried out by using micrometer-sized beam spots which are scanned in an overlapping concentric circle pattern (Dierolf *et al.*, 2010*b* ▶), and one could carry out a ptychographic scan of the specimen with such a beam and then subsequently use a nanofocused beam to record the fluorescence data. However, ptychography has also been demonstrated using a regular raster pattern with a focused beam (Thibault *et al.*, 2008 ▶). This allows one to record ptychographic and fluorescence image data simultaneously and to even use the ptychographic probe reconstruction for deconvolution with the fluorescence image as a way of obtaining higher resolution fluorescence maps (Vine *et al.*, 2012 ▶). Ptychographic imaging methods have seen considerable development, and yield high quality and quantitative phase contrast images and tomograms (Dierolf *et al.*, 2010*a* ▶; Holler *et al.*, 2014 ▶) of the specimen at high resolution, ideal for correlative imaging as described above.

The routine combination of ptychography with X-ray fluorescence microscopy will be greatly enhanced through the use of diffraction-limited storage rings, but several challenges must be overcome. Relatively few pixelated area detectors have sustained image recording rates (frame rates) of greater than 100 Hz, which would be suitable only for pixel transit times of 10 ms or larger and thus not meet the challenges outlined in Table 2 ▶. New pixel array systems such as the Eiger detector from the Paul Scherrer Institute (Johnson *et al.*, 2012 ▶) and Dectris offer burst frame rates of up to 3 kHz, while systems using phosphors for conversion of the X-ray image to visible light which is then imaged onto a high-speed visible-light CMOS detector can acquire small-pixel-count images at burst rates of several hundred kHz. However, ptychographic diffraction patterns cover a large range of scattering angles beyond that of the direct illumination beam, so the detector must have a very high dynamic range in order to successfully record reconstructable diffraction intensities. Phosphor-lens-visible-camera systems tend to have low dynamic range due to parasitic light scattering, so direct X-ray detection is preferred. Furthermore, photon-counting detectors such as the Eiger pixel array detector are not well suited to detecting more than one photon per pixel per electron bunch in the storage ring, so with diffraction-limited storage rings one may want to use analog charge integration rather than photon counting for the brighter regions of the diffraction patttern. Fortunately, fast-frame-rate charge-integrating (Ordavo *et al.*, 2011 ▶; Doering *et al.*, 2011 ▶; Bergamaschi *et al.*, 2011 ▶) and mixed-mode (Vernon *et al.*, 2007 ▶) detectors under development offer such capabilities. Finally, ptychography requires simultaneous phasing of the diffraction pattern from each pixel of a scan, so the combination of ptychography with high-definition X-ray fluorescence nanotomography will involve the reconstruction of staggeringly large datasets.

## 5. Data processing and analysis   

Consider raw data recording for a combined 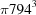 = 1.57 GigaSample tomographic fluorescence and ptychography scan lasting 5 h, where one records 1000 spectral points from a fluorescence detector and a 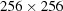 diffraction pattern recording at each pixel. If recorded as uncompressed 32-bit floating point numbers, such a raw dataset would consume 381 TeraBytes of storage space in the 5 h, which exceeds the ∼200 TeraByte storage generated today from all APS beamlines added together over a month. Even if one reaches a modest percentage of the ultimate aggregate performance gains envisioned in Table 2 ▶, it is an understatement to say that simple recording of raw data for later analysis is challenging. In fact, the experimenter is best served by seeing the reconstructed data emerge as the experiment proceeds, so that one can react to surprises in the sample with follow-up scans at higher detail or larger field of view, or by seeing that there were failures in the preparation of this sample so that a new one should be loaded. It is clear that the opportunities created by diffraction-limited storage rings will require corresponding efforts in advancing the processing of data.

X-ray fluorescence tomography involves large data volumes at several locations along the pipeline from acquisition to spectral deconvolution, tomographic reconstruction, and exploration of the resultant three-dimensional datasets. At present, most X-ray fluorescence data are saved to disk for later analysis by least-squares fitting, though the Maia detector system is a notable exception, with spectral deconvolution occurring in hardware using a dynamic analysis method (Ryan, 2000 ▶; Ryan & Jamieson, 1993 ▶) implemented in a field-programmable gate-array based processor (Kirkham *et al.*, 2010 ▶). With enormous pixel rates, least-squares fitting of single-pixel spectra will not keep pace with measurement, and we anticipate that dynamic analysis methods will become more widely used. In addition, high throughput of specimens for 2D analysis will lead to automated approaches for determining the pivotal spectral parameters (elemental species present, energy calibration, scatter profiles, *etc.*) currently required in all approaches. Tomographic reconstruction will hopefully leverage off full-field technologies, which are extremely well developed (Wang *et al.*, 2001 ▶; Mader *et al.*, 2011 ▶; Gürsoy *et al.*, 2014 ▶), so this should not be a bottleneck to development. Volumetric X-ray fluorescence tomography datasets are actually four-dimensional, representing several elemental distributions and scatter signals simultaneously, and will benefit from new approaches in segmentation and exploration.

### 5.1. Alignment   

**Figure 6:**
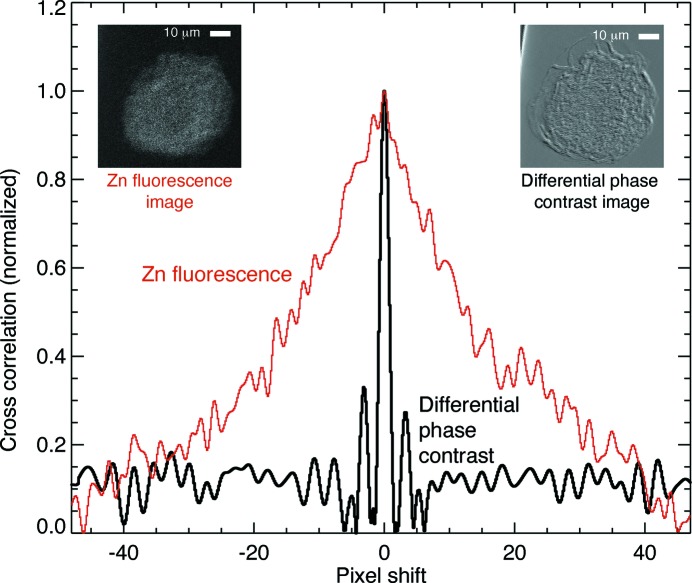
In order to align low-signal fluorescence projections onto a common rotation axis for tomography reconstruction, one can use other stronger signals. Shown here is the cross-correlation between two differential phase contrast images (one is shown at inset, upper right) and two Zn fluorescence images (one is shown at inset, upper left). Even though the simple differential phase contrast image is less easily interpretable than an image of the total projected phase, the cross-correlation peak is dramatically narrower, allowing much more precise alignment from this signal acquired simultaneously with the fluorescence data. The data shown here are for 3 ms dwell-time images of freeze-dried oocytes acquired at beamline 2-ID-E at the APS (Hong *et al.*, 2014 ▶).

Almost all rotation stages show some degree of ‘runout’ error, where the axis of rotation shifts around somewhat with rotation angles. As a result, in X-ray nanotomography one usually needs to determine and then correct for errors in the alignment of individual projections. Fluorescence projections may involve the recording of a relatively small number of photons, with fewer still if dose fractionation is employed (§4.4). This can raise challenges in image alignment, which is already thought to be one of the factors limiting full realisation of dose fractionation (McEwen *et al.*, 1995 ▶). One possibility is to use the larger photon count of phase contrast imaging for alignment of low-dose fast fluorescence images, if both signals are measured simultaneously. This has been explored with reference to two projection images rather than a full rotation series (Hong *et al.*, 2014 ▶), where phase contrast was shown to give significantly sharper cross-correlation peaks compared with the zinc fluorescence signal in mouse oocytes (Fig. 6 ▶). One can also include alignment shifts as a parameter to be optimized globally in iterative reconstruction approaches (Dengler, 1989 ▶; Mayo *et al.*, 2007 ▶) but now using the phase contrast as well as the fluorescence signals.

### 5.2. Self-absorption correction   

**Figure 7:**
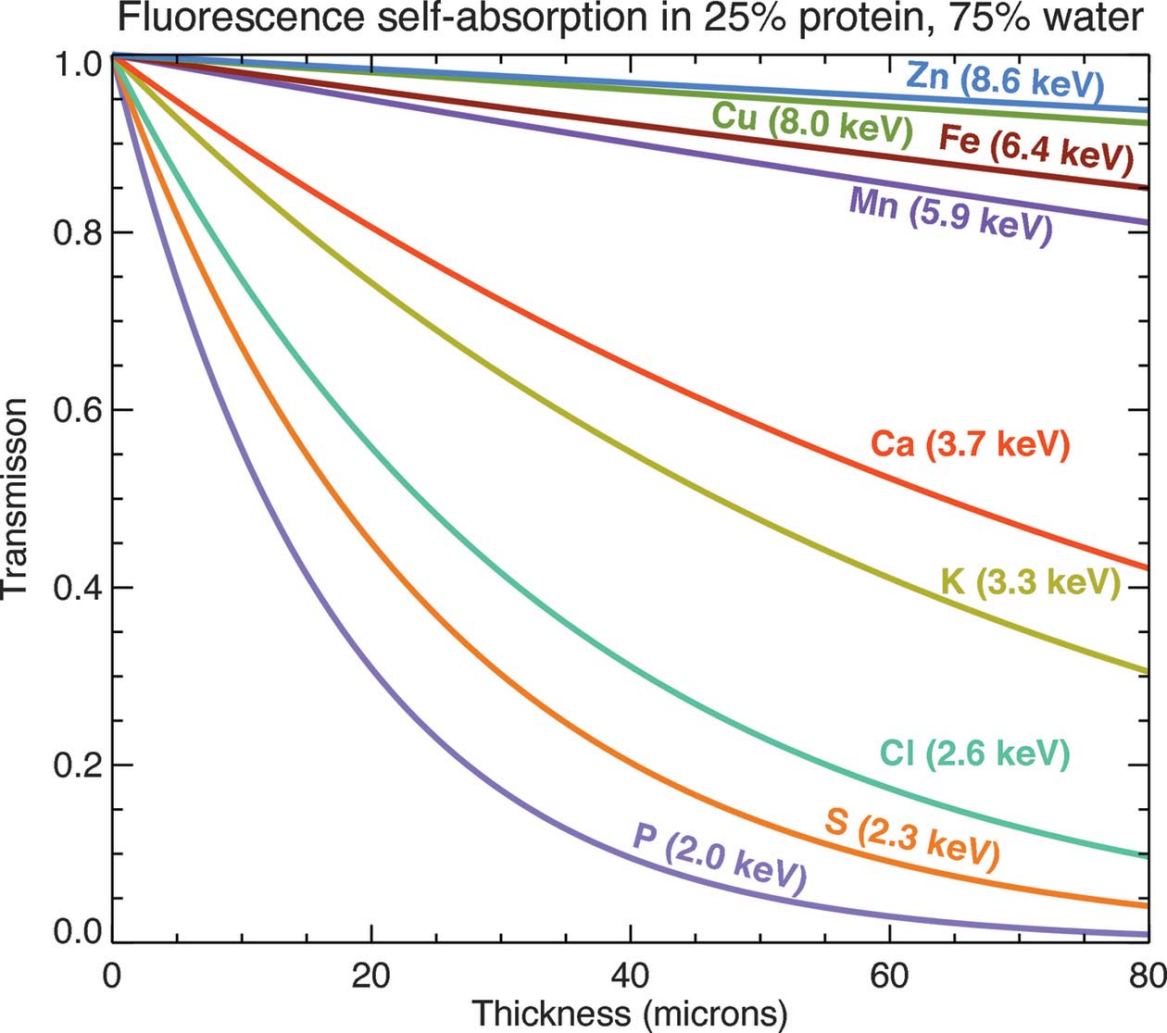
Self-absorption can give rise to errors and artifacts in fluorescence tomography (as well as incorrect ratios of elements) if not corrected. Shown here is the self-absorption calculated for fluorescence from a number of biologically interesting elements due to varying thicknesses of a 25% generic protein/75% water mix representing the cytosol of a typical cell (Luby-Phelps, 2000 ▶).

Because fluorescent X-ray photons from trace elements are emitted at characteristic X-ray energies which can be quite a bit lower than that of the incident beam, some of the fluorescent signal can be reabsorbed along the path from the illumination column to the detector. This changes not only the quantitation but also the apparent ratio between key elements, while also introducing artifacts into the three-dimensional reconstruction because the basic assumptions of linear signal addition along a projection direction are violated. Fig. 7 ▶ shows the transmission of fluorescence from a variety of elements of biological relevance through protein/water modelling mammalian tissue. It is clear that three-dimensional measurements of elements lighter than Ca in hydrated cells (size 10–30 µm) will have to address self-absorption effects.

Several approaches to addressing self-absorption exist. Golosio *et al.* have used a combination of absorption, Rayleigh and Compton scatter signals to apply self-absorption corrections in a mineral specimen (Golosio *et al.*, 2003 ▶). Other correlative measurements (such as the quantitative phase contrast methods discussed in §4.6) could be used in this same manner to provide even more robust estimates of self-absorption and so determine accurate corrections. Iterative approaches have been applied to ‘confocal’ fluorescence X-ray imaging (Vekemans *et al.*, 2004 ▶) where a capillary at 90° to the incident beam is used to collect the fluorescence signal from only a single depth plane at a time (this is not a low-dose approach, since the fluorescence from other depth planes is not collected); this approach is not readily scaled to nano­tomography due to the resolution requirements of the capillary. Iterative self-absorption correction algorithms have also been developed for visible-light fluorescence microscopy (Freiberger *et al.*, 2011 ▶), and are now under active development for X-ray fluorescence tomography.

### 5.3. Pattern recognition: extracting meaning from large datasets   

X-ray fluorescence nanotomography is ultimately not about voxels and photons but about gaining insights into the structure and chemical functioning of the sample under study. In transmission tomography, a wide variety of post-reconstruction analysis methods exist, such as calculations of connectivity for fluid flow through a material, or of surface area in porous catalytical particles. These calculations assume a sharp density difference in the reconstruction to allow one to threshold the volumetric data and find surfaces of various structures; unfortunately, this often requires manual segmentation.

One of the ways in which one can exploit the capabilities of diffraction-limited storage rings is to study not single examples of biological specimens but populations, so that one can gain real statistics on variations that occur in nature. A first example of this approach has been the automatic identification of individual cell types in a mixed sample, allowing one to generate statistical measures of the variation in metal content in cells separated by type (Wang *et al.*, 2014 ▶). One could imagine extending this approach from 2D to 3D, and performing statistical analysis not just on cell types but on organelle types within cells.

## 6. Discussion   

Trace elements play key roles in life and in diseases, so understanding their three-dimensional distribution within cells and tissues is important. Diffraction-limited storage rings offer the potential for transforming X-ray fluorescence nanotomography of cells and tissues from being a heroic experiment executed on the timescale of a day to a rapid measurement method. Such studies can provide insight into the surprising role that trace elements play in normal processes like oocyte development (Kim *et al.*, 2010 ▶), and in debilitating disorders such as Wilson disease (Ralle *et al.*, 2010 ▶). We have outlined here the game-changing nature of diffraction-limited storage rings for X-ray fluorescence tomography, and outlined some of the technical approaches one could consider as well as some of the challenges that lie ahead. Only by addressing these challenges in optics and detectors, in scanning systems and sample preparation and handling, and in computation can one fully exploit new sources, and we feel these challenges should be addressed with just as much effort as is now being brought to bear on accelator physics developments. Taken together, these developments can open up exciting new scientific landscapes.

## Figures and Tables

**Table 1:** Example source parameters for the multi-bend achromat lattice upgrade proposed for the Advanced Photon Source The radiation source size and divergence are calculated for a 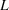 = 4.8 m-long undulator at 10 keV using 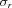 = 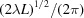 and 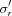 = 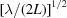 (Onuki & Elleaume, 2003 ▶). As can be seen, the source has about five spatially coherent ‘modes’ in the horizontal, and just over one in the vertical, so that some spatial filtering will still be required in the horizontal direction in particular to achieve a diffraction-limited focus. The product 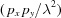 is 5.7 for the upgraded source, compared with 438.1 with 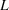 = 2.4 m undulators today, showing nearly a hundredfold increase in source brightness even before undulator optimization and higher beam current are taken into account.

Parameter	Electron beam	Photon beam	Combination	Today
Horizontal size	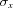 = 21.5 µm	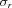 = 5.5 µm	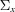 = 22.2 µm	275.0 µm
Horizontal divergence	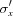 = 3.1 µrad	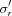 = 3.6 µrad	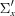 = 4.8 µrad	12.1 µrad
Vertical size	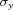 = 4.0 µm	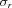 = 5.5 µm	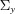 = 6.8 µm	10.7 µm
Vertical divergence	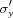 = 1.7 µrad	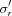 = 3.6 µrad	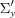 = 4.0 µrad	6.2 µrad
Phase space parameter 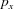	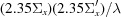 at 10 keV		4.69	148.5
Phase space parameter 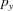	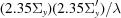 at 10 keV		1.20	2.95
Total modes 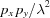 at 10 keV			5.7	438

**Table 2:** Potential performance gain factors in X-ray fluorescence nanoprobe analysis that could be expected from a diffraction-limited storage ring along with improvements in spectral bandpass (such as the use of a multilayer monochromator instead of a crystal monochromator), improved nanofocusing optics (such as stacked, high-aspect-ratio zone plates), and improved detector solid angle (such as is offered by the Maia detector) The total detected flux includes elastic and Compton scattered photons, while the elemental flux represents the signal in a typical elemental fluorescence line (such as from Fe or Mn) from representative cells. The baseline for these estimates is the operating experience of the Bionanoprobe at the Advanced Photon Source (Chen *et al.*, 2014 ▶), assuming pixel transit times in that instrument of 100 ms for detecting about 100 photons in typical elemental fluorescence lines. It may be difficult to realise the exact gain factors listed here, either separately or especially in aggregate, but it is still instructive for considering future possibilities.

Improvement	Potential improvement factor	Focused flux ( 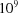 s 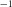 )	Total detected flux ( 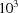 s 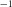 )	Elemental flux ( 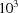 s 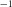 )	Pixel time (µs)
Bionanoprobe today	1	3.5	30	1.0	100000
Diffraction-limited storage ring	150	525	4500	150	667
Increased spectral bandpass	10	5250	45000	1500	67
Improved nanofocusing optics	3	21000	180000	6000	22
Improved detector solid angle	1.8	37800	324000	10800	12
